# Newly Discovered Adipokines: Pathophysiological Link Between Obesity and Cardiometabolic Disorders

**DOI:** 10.3389/fphys.2020.568800

**Published:** 2020-09-02

**Authors:** Jung A. Kim, Kyung Mook Choi

**Affiliations:** Division of Endocrinology and Metabolism, Department of Internal Medicine, College of Medicine, Korea University, Seoul, South Korea

**Keywords:** adipokine, pathophysiology, cardiometabolic disorders, adipocyte, obesity

## Abstract

With the increasing prevalence of obesity, obesity-related problems such as cardiometabolic disorders (CMD), are also rapidly increasing. To prevent and alleviate the progressive course of CMD, it is important to discover the pathophysiological mechanisms between obesity and CMD. Adipose tissue is now recognized as an active endocrine organ that releases adipokines. Adipokines play a pivotal role in chronic low-grade inflammation, oxidative stress, and impaired insulin signaling, contributing to metabolic derangement and leading to CMD. Recent studies have provided substantial evidence supporting the association between adipokines and CMD. In this review, we highlight the pathophysiological action of adipokines in CMD that includes metabolic syndrome, type 2 diabetes, non-alcoholic fatty liver disease, and cardiovascular diseases. We focused on translational and clinical research of novel adipokines associated with metabolic and cardiovascular regulation. Exploration of the role of these adipokines connecting obesity and CMD may provide a perspective on adipokine-based therapeutic implications for CMD.

## Introduction

**FIGURE 1:**
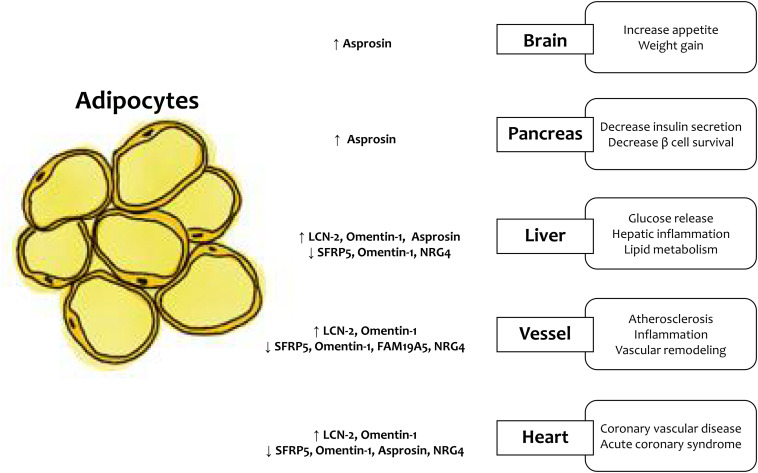
LCN-2, lipocalin-2; SFRP5, secreted frizzled-related protein 5; NRG4, neuregulin 4.

According to the WHO, the prevalence of being overweight and obesity has doubled since 1980, and ~ 30% of adults are either overweight or obese (Chooi et al., 2019). Increased fat accumulation leads to adverse health outcomes; thus, obesity imposes a significant health challenge. From the perspective of the cardiometabolic disorder (CMD) development, obesity is considered a major pathophysiological factor involved in the exaggeration of chronic systemic inflammation, oxidative stress, endothelial dysfunction, and energy homeostasis (Marseglia et al., 2014). Excess adipose tissue accumulation caused by over nutrition and physical inactivity induces adipocyte dysfunction. In addition, excess adipose tissue accumulates as ectopic fat in the liver, vasculature, muscle, and heart (Britton and Fox, 2011). Recently, adipose tissue has been regarded as an endocrine organ secreting cytokines called adipokines, and the secretion process of endocrine factors is now a topic of interest (Kershaw and Flier, 2004). The expression of adipokines, which have anti-inflammatory or pro-inflammatory activities, varies depending on the site and distribution of adipose tissue accumulation. The dysregulation of adipokines induces systemic inflammation and contribute to the obesity-related metabolic complications, such as metabolic syndrome, type 2 diabetes (T2DM), non-alcoholic fatty liver disease (NAFLD), atherosclerosis, and cardiovascular disease (CVD). In this review, we summarize the pathophysiological actions of newly discovered adipokines (Table 1 and Figure 1).

**TABLE 1:** Actions of newly discovered adipokines in cardiometabolic disorders (CMD).

Adipokine	Action on CMD	Metabolic syndrome	Type 2 diabetes	NAFLD	CVD
Lipocalin-2	Harmful	↑ Triglyceride ↑ Insulin resistance ↑ Metabolic syndrome	↑ Glucose ↑ Diabetes	↑ Hepatic inflammation ↑ Hepatic lipid droplet accumulation, apoptosis ↑ Hepatic fibrosis	↑ Atherosclerosis ↑ Coronary heart disease
SFRP5	Protective	↓ HOMA-IR ↓ Lipid profile	↓ Impaired glucose tolerance ↓ Type 2 diabetes ↑ GLP-1 agonist	↓ Hepatic inflammation ↓ NAFLD progression	↓ Coronary heart disease onset and severity
Omentin-1	Controversy	↓ Waist circumference ↓ HOMA-IR ↑ HDL	↑↓Controversy	↑ NAFLD ↑ Hepatocyte ballooning	↑↓ Controversy ↓ Arterial stiffness and carotid plaque ↑ Cardiovascular disease
Asprosin	Controversy	↑ Insulin resistance	↑ Type 2 diabetes ↑ Risk of type 2 diabetes	–	↓ Angina ↓ Acute coronary syndrome
FAM19A5	Protective	–	↑ Hyperglycemia ↑ Type 2 diabetes	–	↓ Vascular smooth muscle cell proliferation ↑ Pulse wave velocity
Neuregulin 4	Protective	↓ Insulin resistance	↓ Type 2 diabetes	↓ Hepatic steatosis ↓ NAFLD progression	↓ Atherosclerosis ↓ Severe coronary artery lesion

*CMD, cardiometabolic disorder; NAFLD, non-alcoholic fatty liver disease; CVD, cardiovascular disease; SFRP5, secreted frizzled-related protein 5; HOMA-IR, homeostatic model assessment-insulin resistance; HDL, high-density lipoprotein. ↑, increase; ↓, decrease.*

## Lipocalin-2 (LCN-2)

The action of LCN-2 is complex, on the one hand LCN-2 is upregulated in cases of insulin resistance such as obesity and diabetes and on other hand LCN-2 has a protective effect on NAFLD.

### Preclinical Studies

LCN-2 is a 25 kDa protein that is also known as neutrophil gelatinase-associated lipocalin (NGAL) (Kjeldsen et al., 1993). LCN-2 has been studied for its role in the innate immune response to bacterial infection (Flo et al., 2004) and as a reliable marker of acute kidney injury of diverse etiologies. LCN-2 is expressed in various sites, such as liver, kidney, brain, lung, and adipocytes (Asimakopoulou et al., 2016). Inflammatory stimuli, including nuclear factor-kB (NF-kB) pathway activation, induce LCN-2 expression (Jayaraman et al., 2005), and that the expression of LCN-2 is abundant in adipocytes and macrophages. In studies using an animal model, the expression of LCN-2 in adipose tissue and liver, and its concentration was higher in diabetic/obese mice than that in lean mice (Wang et al., 2007). The adipose-specific expression of LCN-2 is dependent on CCAAT-enhancer-binding protein (C/EBP) trans-activation in the LCN2 promoter (Yan et al., 2007). LCN-2 deficient mice show insulin-sensitive metabolic properties by inhibition of arachidonate 12-lipoxygenase, which is related to insulin resistance and inflammation (Law et al., 2010). LCN-2 expression was induced by agents promoting insulin resistance and was suppressed by insulin-sensitizing anti-diabetes drug (Yan et al., 2007). Besides, LCN-2 is related to the development and progression of fatty liver disease. LCN-2 was upregulated in the liver with non-alcoholic steatohepatitis (NASH), especially around inflammatory cell clusters (Semba et al., 2013). In acute liver damage and inflammation, LCN-2 was also upregulated, and its expression was correlated with hepatic inflammation (Borkham-Kamphorst et al., 2013). LCN-2 protects against diet-induced NAFLD by regulating lipolysis, fatty acid oxidation, *de novo* lipogenesis, lipid peroxidation, and apoptosis (Xu et al., 2019). Based on these results, LCN-2 has a hepatoprotective effect and might have a role as a putative marker of NAFLD and NASH. Recently, LCN-2 was identified as a novel osteokine, bone-derived hormone, and suppressor of appetite via melanocortin 4 receptor (MCR4) (Mosialou et al., 2017).

### Clinical Studies

LCN-2 concentration had a positive correlation with several adiposity variables, triglyceride level, glucose level, high sensitivity C-reactive protein (hs-CRP) concentration, and homeostatic model assessment-insulin resistance (HOMA-IR) (Wang et al., 2007). The LCN-2 concentration was significantly higher in metabolic syndrome patients than that in controls (Curro et al., 2020). Therefore, LCN-2 is a marker of obesity-induced metabolic derangement. Recently, metabolic syndrome has been regarded as a risk factor for incident vascular dementia. For the differential diagnosis of vascular dementia, the concentration of not only serum LCN-2 but also that of cerebrospinal fluid LCN-2 emerged as a novel biomarker (Llorens et al., 2020). LCN-2 was highly expressed in human atherosclerotic lesions and mouse models of atherosclerosis and myocardial infarction (Hemdahl et al., 2006). We previously reported that circulating LCN-2 is positively associated with coronary heart disease independent of associated metabolic factors (Choi et al., 2008). These findings indicate that the circulating LCN-2 concentration could be a useful surrogate to evaluate the effect of a potential therapeutic intervention for CMD.

## Secreted Frizzled-Related Protein 5 (SFRP5)

SFRP5 is an anti-inflammatory adipokine and it is an endogenous inhibitor of the wingless-type family member 5A (Wnt5a) signaling pathway.

### Preclinical Studies

The imbalance of Wnt5a and SFRP5 increases adipose tissue inflammation and insulin resistance (Ouchi et al., 2010). Obesity and adipocyte inflammation increase in the ratio of pro-inflammatory Wnt5a to anti-inflammatory SFRP5. Overexpression and secretion of SFRP5 in 3T3-L1 adipocytes may be a major pathophysiology of insulin sensitizers (Lv et al., 2012). SFRP5 over-expression via adenovirus could alleviate obesity, adipose inflammation, and hepatosteatosis (Ouchi et al., 2010). In an experiment with NASH mouse model, SFRP5 significantly improved liver inflammation and fatty lesion scores (Chen et al., 2017). Moreover, SFRP5 intervention led to the downregulation of pro-inflammatory cytokines, including interleukin (IL)-1β, IL-6, tumor necrosis factor-α (TNF-α), and monocyte chemoattractant protein-1, in the liver. The expression of Wnt5a in endothelial cells and smooth muscle cells, is involved in the development of atherosclerosis (Cheng et al., 2008). Compared to wild type mice on a regular diet, high-fat diet apo E −/− mice showed a higher expression of Wnt5a in macrophage-rich regions of plaques. In the human atherosclerotic plaque, the expression of Wnt5a was correlated with the expression of CD68, which is a marker of macrophages. Therefore, Wnt5a is upregulated in murine and human macrophages and exerts action as an effector of macrophages (Christman et al., 2008). Moreover, ox-LDL triggers atherosclerosis by increasing the expression of Wnt5a in human macrophages and differentiated THP-1 cells. Furthermore, Wnt5a has a role in arterial calcification (Woldt et al., 2012). In recent study, restored Wnt5a-induced endothelial dysfunction through the JNK pathway, as well as upregulated nitric oxide (NO) production (Cho et al., 2018). Therefore, as an inhibitor of the Wnt5a signaling pathway, SFRP5 might have a pivotal role in CVD.

### Clinical Studies

Overweight/obese subjects have significantly lower SFRP5 levels than normal-weight subjects (Hu et al., 2013). Furthermore, SFRP5 levels correlated to body mass index, waist-to-hip ratio, percent body fat, HOMA-IR, and lipid profiles. Caloric restriction increases SFRP5 levels in obese patients (Schulte et al., 2012), whereas surgically induced weight loss decreases circulating Wnt5a levels (Catalan et al., 2014). SFRP5 levels independently decrease in patients with impaired glucose tolerance and newly diagnosed T2DM (Hu et al., 2013). The concentration of SFRP5 decreases in hyperglycemia but increases with the administration of the GLP-1 agonist, liraglutide (Hu et al., 2013). In a study on obese women, SFRP5 levels showed a tendency to decrease with NAFLD progression. In addition, hepatic SFRP5 protein levels were significantly lower in NASH than in control groups (Gutierrez et al., 2015). Wnt5a in the serum of atherosclerotic subjects was higher than that in the serum of normal subjects (Malgor et al., 2014). The severity of atherosclerotic plaque is correlated with the expression of Wnt5a. Subjects with coronary heart disease (CHD) had significantly lower levels of SFRP5 than non-CHD controls, and the serum level of SFRP5 was negatively associated with the severity of CHD (Miyoshi et al., 2014). They suggested that low SFRP5 levels may contribute to CVD. Considering the role of SFRP5 as an inhibitor of the Wnt5a pathway, increased plasma Wnt5a accompanied by reduced circulating SFRP5 in patients with CVD compared to that in non-CVD controls after adjusting for age, sex, and BMI (Akoumianakis et al., 2019).

## Omentin-1

Omentin-1, an adipokine secreted from visceral adipose tissue, modulates insulin resistance, and has anti-inflammatory and anti-atherosclerotic effects.

### Preclinical Studies

Omentin-1, also known as intelectin-1, is a novel visceral fat depot-specific secretory glycoprotein (Tsuji et al., 2001). The omentin-1 gene is located in the 1q22-q23 chromosomal region, which has been linked to T2DM in several populations (Xiang et al., 2004). In an experimental study, omentin-1 mRNA was predominantly expressed in visceral adipocytes and scarcely expressed in subcutaneous fat in humans and monkeys (Yang et al., 2006). Omentin-1 ameliorates insulin resistance, inflammation, and atherosclerosis via AMP-activated protein kinase (AMPK)/Akt/NF-kB/mitogen-activated protein kinase pathways (Watanabe et al., 2017). *In vitro*, addition of recombinant omentin-1 increased Akt phosphorylation and improved glucose uptake in both subcutaneous and omental human adipocytes (Yang et al., 2006). Insulin and glucose are associated with a significant dose-dependent decrease in omentin-1 mRNA expression, protein expression, and secretion into conditioned medium. Additionally, hyper-insulinemic induction in healthy subjects significantly reduced plasma omentin-1 levels (*P* < 0.01). The role of omentin-1 in the pathogenesis of CVD has been studied. A 4-week infusion of omentin-1 into Apo E−/− mice delayed the development of aortic atherosclerosis. Omentin-1 levels were significantly reduced in coronary endothelium and epicardial fat (Watanabe et al., 2016).

### Clinical Studies

The gene expression and plasma levels of omentin-1 were significantly higher in lean subjects than in obese and overweight subjects (de Souza Batista et al., 2007). Plasma omentin-1 levels have a negative correlation with BMI, waist circumference, leptin levels, and HOMA-IR and a positive correlation with adiponectin and HDL levels. Omentin-1 levels were found to be significantly decreased in T2DM compared to those in the normal glucose control (Yoo et al., 2011). However, other studies showed that omentin-1 levels were higher in T2DM patients than in controls (Herder et al., 2017; Hayashi et al., 2019). According to a recent meta-analysis based on 42 studies, lower omentin-1 concentrations were observed in gestational diabetes mellitus and T2DM than that in controls (Pan et al., 2019). Moreover, circulating omentin-1 levels decreased in diabetic patients with microvascular complications, such as retinopathy, nephropathy, and neuropathy (Tekce et al., 2014; Jung et al., 2015; Wan et al., 2015). Despite the positive relationship between insulin resistance and NAFLD, an unexpected paradoxical increase in omentin-1 in subjects with NAFLD was observed. Serum levels of omentin-1 were significantly higher in biopsy-proven NAFLD than in the healthy control (Yilmaz et al., 2011). Moreover, serum omentin-1 levels were related to the degree of hepatocyte ballooning, independent of risk factors, including metabolic parameters. In the genetic study, omentin-1 rs2274907(326A/T) polymorphisms were significantly associated with NAFLD (OR: 2.30, 95% CI:1.30–3.80, *P* = 0.003); omentin-1 polymorphism could be a candidate for predisposition to NAFLD (Kohan et al., 2016). In a cross-sectional study, omentin-1 was inversely related to the arterial stiffness and carotid plaque in patients with T2DM. Circulating omentin-1 was found to be an independent pivotal factor for the presence of carotid plaque in patients with T2DM, regardless of other cardiovascular risk factors and medication history, including statin therapy (Yoo et al., 2011). On the contrary, prospective studies suggested that omentin-1 is a cardiovascular risk factor. The European Prospective Investigation into Cancer and Nutrition (EPIC)-Potsdam study showed that higher levels of omentin-1 were related to an increased risk of stroke in metabolically healthy participants (Menzel et al., 2016). Recent 14 years of follow-up in a prospective study on diabetes without a previous cardiovascular event showed that higher omentin-1 concentrations are associated with a higher risk for CVD events, incidence of primary stroke, and incidence of cardiovascular death even after adjustment for cardiovascular risk factors (Niersmann et al., 2020). As mentioned above, a highly heterogeneous association between omentin-1 and CMD has been reported. These discrepancies might have originated from different study populations and regions or might be attributed to the compensatory mechanism of omentin-1 to protect against CMD.

## Asprosin

Asprosin is a novel adipokine which involves with appetite, glucose homeostasis, and insulin resistance.

### Preclinical Studies

Asprosin is a fasting-induced glucogenic protein hormone that was discovered as an adipokine from patients with neonatal progeroid syndrome (NPS) (Romere et al., 2016). NPS patients show low asprosin levels and have an extremely lean body shape regardless of insulin action. In a study with asprosin-deficient model mice were protected from obesity and diabetes after being administered a high-fat diet for 6 months (Duerrschmid et al., 2017). Asprosin has multiple effects on the central nervous system and peripheral tissues, such as heart, adrenal, lung, white fat, and kidney, and regulates appetite, glucose homeostasis, and insulin resistance (Romere et al., 2016). The role of asprosin in the central nervous system is mediated by its action on anorexigenic pro-opiomelanocortin (POMC) neurons and the orexigenic agouti-related peptide (AgRP) neurons after crossing the blood–brain barrier. These mechanisms lead to increased food intake, regulated energy homeostasis, and a tendency for adipose accumulation and increased body weight (Duerrschmid et al., 2017). White adipose tissue-secreted asprosin modulates hepatic glucose release through OR4M1 (Li et al., 2019). In the liver, asprosin activates the G protein-cAMP-PKA pathway and elevates the circulating glucose level. Persistent high levels of glucose and obesity-induced free fatty acids induce oxidative stress and a high level of chronic inflammation, which contributes to pancreatic islet cell dysfunction and apoptosis. Asprosin could bind to Toll-like receptor 4 (TLR4), stimulate the TLR4/c-JNK-mediated pathway, and increase ROS production and levels of pro-inflammatory cytokines, leading to the apoptosis of β cells (Lee et al., 2019). *In vitro* and *in vivo* experiments showed that glucose serves as a negative regulator of plasma asprosin in a feedback loop. Considering the dual action of asprosin as a glucogenic and orexigenic molecule, asprosin is a potential therapeutic target of obesity-related CMD. Anti-asprosin monoclonal antibodies reduce appetite, blood glucose concentration, and body weight (Romere et al., 2016). An *in vitro* study showed that asprosin prevents high glucose condition-induced cardiomyocyte apoptosis by reducing oxidative stress (Feng et al., 2018).

### Clinical Studies

Asprosin is mainly secreted by adipose tissue; obese subjects have a higher concentration of asprosin than control groups (Duerrschmid et al., 2017). In a study with 117 patients undergoing bariatric surgery, the pre-operative level of serum asprosin was related to weight reduction at 6 months after bariatric surgery (Wang et al., 2019). In addition, several studies presented a close relationship between asprosin and insulin resistance in human. In a cross-sectional study, the circulating asprosin levels were significantly higher in patients with T2DM and positively correlated with the risk of T2DM (Zhang et al., 2019). Plasma asprosin levels were increased in patients with glucose dysregulation and correlated with insulin resistance and first-phase insulin secretion (Wang et al., 2018b). Interestingly, asprosin is a novel marker for angina pectoris and the severity of acute coronary syndrome (Acara et al., 2018).

## FAM19A5

The family with sequence similarity 19[chemokine (C-C motif)-like] member A5 (FAM19A5), also called TAFA5, a newly identified adipokine (Wang et al., 2018a), has a protective effect from CMD. The expression of FAM19A5 is upregulated in lipopolysaccharide-stimulated monocytes and macrophages; thus, it might be involved in the regulation of peripheral immune cell activity (Wang et al., 2015). In a rodent model, FAM19A5 inhibited vascular smooth muscle cell proliferation, migration, and post-injury neointima formation (Wang et al., 2018a). FAM19A5 is profoundly expressed and released from adipocytes of lean mice, and this expression is markedly decreased in obesity. The expression of FAM19A5 is reduced by TNF-α in human adipocytes, which means that obesity-induced inflammatory cytokines may cause the downregulation of FAM19A5 (Tourniaire et al., 2013). In contrast to the protective role of FAM19A5, another study showed an inverse correlation between FAM19A5 and cardiometabolic risk factors. In a cross-sectional study, the serum FAM19A5 level was higher in subjects with T2DM than in subjects with normal glucose tolerance (Lee et al., 2019). Furthermore, FAM19A5 concentration was positively correlated with abdominal obesity, fasting plasma glucose, HbA1C, and mean pulse wave velocity. Despite the contradictory results between studies, FAM19A5 may have a potential role in obesity-related CMD, such as T2DM and atherosclerosis.

## Neuregulin 4 (NRG4)

NRG4 is a brown adipose tissue secreted adipokine which has beneficial effect on metabolic diseases. NRG4 is a member of the epidermal growth factor family of extracellular ligands, members of which are highly expressed in brown adipose tissue (BAT) (Wang et al., 2014). Nrg4-deficient mice (Nrg4−/−) with more body weight developed more marked hepatic steatosis and insulin resistance compared with controls upon high-fat feeding. In contrast, NRG4 over-expression in adipose tissue was related to less fat accumulation and insulin sensitivity. NRG4 transduces signals through the receptor tyrosine-protein kinases/human epidermal growth factor receptors (ErbB/HER) ErbB3 and ErbB4. The activated ErbB3 and ErbB4 signals have a negative impact on *de novo* lipogenesis in the liver (Wang et al., 2014). In addition, NRG4 signaling is related to disease progression from NAFLD to NASH (Guo et al., 2017). In the diet-induced mouse model with NASH, the expression of NRG4 was decreased, and NRG4 deficiency was related to liver injury, inflammation, and fibrosis. In a human study, subcutaneous WAT NRG4 mRNA levels were negatively related to the percentage of body fat mass and liver fat content (Wang et al., 2014). Subjects with T2DM had reduced adipocyte NRG4 expression than those with normal glucose tolerance. In addition, NRG4 and coronary artery disease (CAD) have a negative association (Tian et al., 2019). The underlying physiology of the relationship between NRG4 and CAD is not fully studied; NRG4 has an anti-apoptotic effect on endothelial cells and prevents the development of atherosclerosis (Sato and Minatsuki, 2019).

## Discussion

Obesity-related altered secretion of adipokines related to low-grade inflammation, oxidative stress, insulin resistance, and β-cell dysfunction has an important role in the development and progression of CMD. To date, substantial experimental and clinical studies have been conducted to elucidate the role of adipokines in CMD, and inconsistent results have been reported. More research using gene analysis, intervention, and adipokine-targeting antibodies are needed to discover the pleiotropic effect of adipokines in CMD. Considering that obesity and CMD are the leading causes of the global burden of mortality, the identification of adipokines involved in the pathophysiologic mechanism associated with CMD may provide an opportunity to prevent deleterious consequences resulting from excess adipose tissue accumulation.

## Author Contributions

JK wrote the first draft of the manuscript. KC contributed to the revision of the manuscript and approved the submitted version of the manuscript. Both authors contributed to the article and approved the submitted version.

## Conflict of Interest

The authors declare that the research was conducted in the absence of any commercial or financial relationships that could be construed as a potential conflict of interest.
